# Supplementary figures and images for: Closing target trimming and CTTdocker programs for discovering hidden superfamily loci in genomes
Source: PLoS One. 2019 Jul 2;14(7):e0209468. doi: 10.1371/journal.pone.0209468 (PMC6605638; doi:10.1371/journal.pone.0209468)

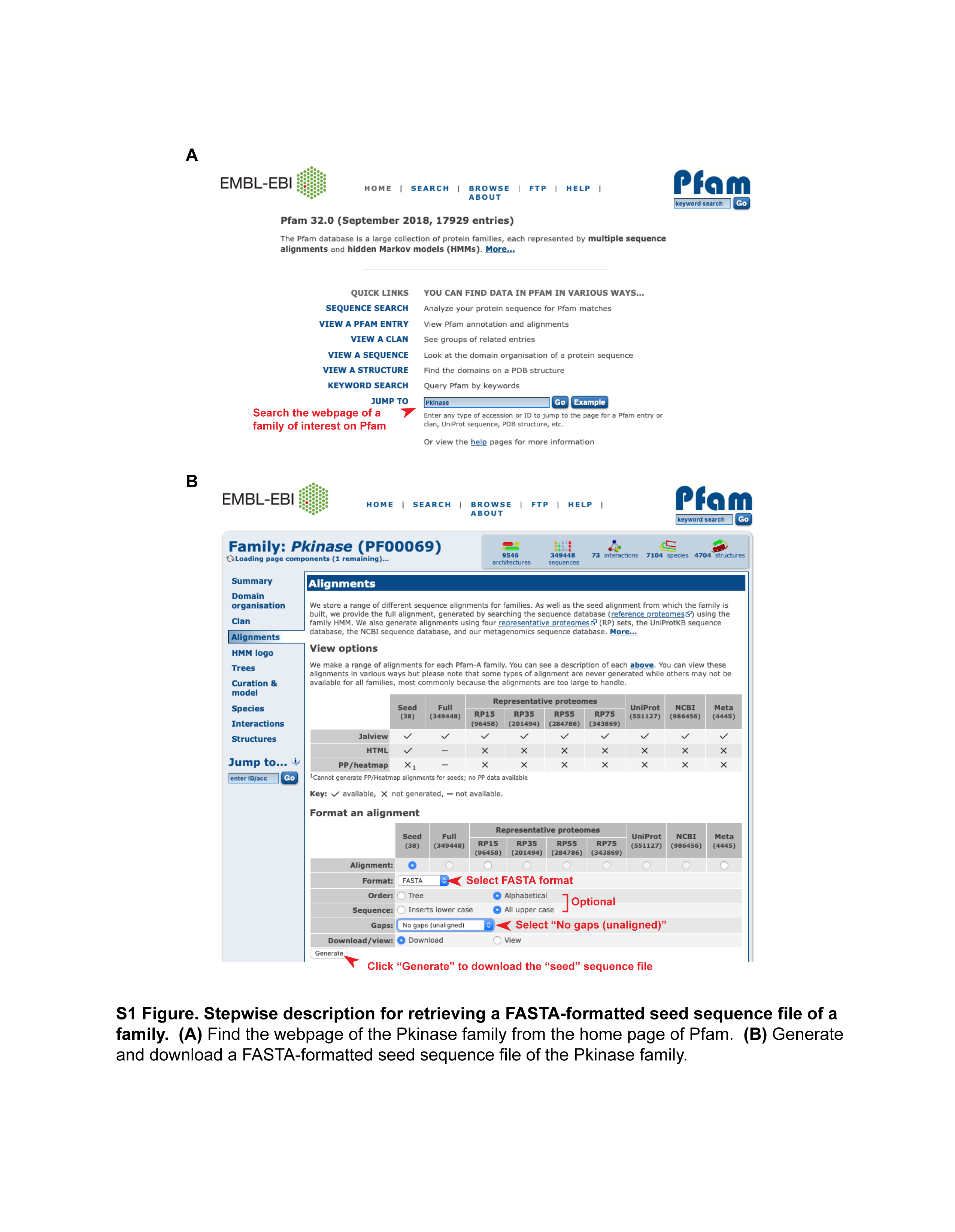

Supplement: S1 Fig — (TIF) [file pone.0209468.s002.tif]
